# Cellularized Thermoformed Scaffolds with Human Septal Chondrocytes Support In Vivo Cartilage Maturation for Auricular Reconstruction

**DOI:** 10.3390/jfb17090453

**Published:** 2026-09-07

**Authors:** Emma Muiños-López, Iñigo Arroyo, Olatz Guaresti, Uzuri Urtaza, Arantza Barco-Martín, Asier Ullate-Agote, Tania López-Martínez, Ane M. Zaldua, Manuel M. Mazo, Froilán Granero-Moltó, Bernardo Hontanilla

**Affiliations:** 1 Experimental Orthopedics Laboratory, Department of Orthopedic Surgery and Traumatology, Clínica Universidad de Navarra (CUN), Pío XII, 36, 31008 Pamplona, Spain; talopez@unav.es (T.L.-M.); fgranero@unav.es (F.G.-M.); 2 Hematology and Cell Therapy Area, Clínica Universidad de Navarra (CUN), Pío XII, 36, 31008 Pamplona, Spain; aullatea@unav.es (A.U.-A.); mmazoveg@unav.es (M.M.M.); 3 Instituto de Investigación Sanitaria de Navarra (IdiSNA), Irunlarrea, 3, 31008 Pamplona, Spain; 4 Bioengineering Program, Technological Innovation Division, Cima Universidad de Navarra, Pío XII 55, 31008 Pamplona, Spain; 5 Department of Plastic Aesthetic and Reconstructive Surgery, Clínica Universidad de Navarra (CUN), Pío XII, 36, 31008 Pamplona, Spain; iarroyop@unav.es; 6 Leartiker S. Coop., Xemein Etorbidea, 12A, 48270 Markina-Xemein, Spain; oguaresti@leartiker.com (O.G.); uurtaza@leartiker.com (U.U.); abarco@leartiker.com (A.B.-M.); amzaldua@leartiker.com (A.M.Z.)

**Keywords:** anotia, septal nasal chondrocytes, ear reconstruction, poly(lactide-b-ethylene glycol) scaffolds, thermoformed, 3D biofabrication, transcriptomics

## Abstract

**Introduction**: The clinical translation of biomaterial-based strategies for ear reconstruction remains limited by several challenges, including scaffold design, selection of an optimal cell source, and stability of the new cartilage tissue. Most recent studies still rely on animal-derived cells and fail to demonstrate the long-term maintenance of the chondrogenic phenotype. **Methods**: In this study, we developed an off-the-shelf poly(lactide-b-ethylene glycol) (PLA-PEG) scaffold combined with clinical-grade alginate (Alg) and cellularized with human nasal chondrocytes for auricular cartilage engineering. Cellularized constructs were evaluated using histological and immunofluorescence analyses to assess extracellular matrix production and quality. In parallel, constructs retrieved after 12 weeks of in vivo implantation were mechanically characterized using a UniVert CellScale testing system to determine variations in compressive strength. Finally, the chondrogenic maturation of the new tissue was evaluated through bulk RNA transcriptomic profiling. **Results**: Histological, immunofluorescence, and transcriptomic analyses demonstrated robust cartilage matrix deposition with improved compressive properties and high expression of chondrogenic markers, such as ACAN, PRG4, and COL2A1. Furthermore, positive staining for the human-specific Ku80 antibody confirmed the presence of cells of human origin in the newly formed tissue after implantation. **Conclusions**: Overall, this study provides evidence that the combination of a PLA-PEG + Alg scaffold with human nasal septal chondrocytes represents a promising strategy for future reconstructive applications in auricular tissue engineering.

## 1. Introduction

Auricular reconstruction for anotia, microtia, or traumatic pinna loss remains among the most challenging surgical procedures in Plastic Surgery. Current available therapeutic strategies rely on three different approaches: Silicone prosthetic ears [1], synthetic material implants [2], and autologous costal cartilage grafts [3]. Modern silicone prosthetic ears use various implant-retained fixation systems, such as magnets, O-ring attachments, or small titanium rods placed in the bone behind the ear, offering a stable and safe retention without adhesives and preventing secondary reconstructive surgeries [4]. However, daily prosthesis removal leads to material deformation, impacting functional longevity.

Synthetic internal implants provide more favorable esthetic outcomes, offering a more realistic appearance. These are strong and stable structures that effectively preserve the ear morphology, such as MedPore^®^, the most utilized polyethylene material in ear reconstruction. Their porous nature allows tissue ingrowth and vascularization, allowing reconstruction in a single surgical stage, even at an early age. Recent reviews, however, determined that MedPore^®^ carries a 15% complication rate after surgery compared to a 2% rate for autologous techniques [5]. Those complications are primarily esthetic, such as an asymmetry of the reconstructed ear or early or strong host immune reactions, such as mechanical encapsulation, chronic sterile inflammation, infections, and extrusion [6,7]. Consequently, the autologous costal cartilage implantation, featuring an absence of foreign-body rejection and lower infection rate, has remained the gold standard for over six decades.

Surgical protocols to perform the autologous costal cartilage reconstruction can be divided into those techniques developed by Brent, Nagata, and Firmin’s modification of the Nagata technique [8]. All require high experience and meticulous handcraft skills to achieve optimal quality esthetic results and satisfactory outcomes [9]. In addition, autologous grafting presents two major drawbacks: significant surgical donor-site morbidity, requiring several costal segments to create a single auricle, and unpredictable auricular morphology. These limitations emphasize the urgent need for alternative approaches.

Tissue engineering is leading a paradigm shift in reconstructive medicine. In the field of Plastic Surgery during the 90’s, pioneering work by Aigner et al., Bujia et al., and Cao et al. demonstrated the feasibility of using cellularized scaffolds shaped as a human ear seeded with human cells [10,11]. Different technological breakthroughs in personalized biofabrication to mimic patient-shape- and size-specific organs, including multi-nozzle 3D bioprinters with temperature control systems [12,13] and enhanced knowledge on the cell–material interface [14], have positioned 3D bioprinting as a useful tool for auricular reconstruction [15,16]. Moreover, continuous improvement in material biocompatibility and degradability reinforce the idea of integrating this technology for clinical applications.

Yet, clinical translation is still limited by important hurdles intrinsic to the auricular architecture. A major obstacle is preserving high anatomical fidelity of the ear over time. The ideal scaffold should provide the intended shape and support while being resorbable in the mid to long term. Polycaprolactone (PCL), poly (glycolic acid) (PGA), and polymers poly (lactic acid) (PLA) (PGA/PLA) scaffolds have demonstrated greater consistency for this purpose [17,18]. Historically, porous high-density polyethylene (pHDPE) (Medpore^®^) has been used in clinical applications. Currently, PCL is taking the lead as a final product material, as it fulfills the whole standards procedures in terms of ISO 10993 [19]. Another critical hitch is to identify the proper cell source capable of expanding efficiently while maintaining robust chondrogenic potential. Numerous studies have explored the use of mesenchymal stem cells from different sources such as adipose tissue, bone marrow, perichondrium, and cartilage progenitor cells [20,21,22]. Although these cell types have shown capacity to generate cartilage tissue in vitro, the translation into in vivo models showed limited results, even when implementing pre-maturation steps or co-culture with chondrogenic stimuli in in vitro pre-implantation. The inadequate quality of the tissue and the procedure complexities limit their clinical applicability.

In this study, we present the development of an off-the-shelf poly(lactide-b-ethylene glycol, PLA-PEG) scaffold combined with clinical-grade alginate (Alg) and cellularized with human nasal chondrocytes (NC) to assess its translational potential. Furthermore, we employed transcriptomic profiling following in vivo implantation to evaluate the maturation of the newly developed tissue. The primary goal of this work is to engineer a fully cellularized ear construct that undergoes effective in vivo maturation, demonstrated with advanced transcriptomic techniques, paving the way for future clinical translation.

## 2. Materials and Methods

### 2.1. Fabrication of Poly(lactide-b-ethylene glycol) Scaffolds

Square scaffolds of 1 × 1 cm^2^ were fabricated to study the initial material response in ectopic models to generate cartilaginous matrix followed by histological, gene expression, and mechanical characterization. The manufacturing technique was fused filament fabrication (FFF) using a TUMAKER NX printer (Tumaker, Oiartzun, Spain) equipped with a direct-drive extrusion system and a 200 μm nozzle. Medical-grade PLA-PEG copolymer (Resomer^®^ LRP t7046, Evonik, Essen, Germany), consisting of a PLA-PDLA-PEG block copolymer with a 70/30 L/D lactide ratio and 4% PEG content, was processed into filament prior to printing. Scaffolds were printed using a double-offset architecture with 47% infill density, previously identified as the most suitable configuration for cartilage tissue engineering applications [23]. Printing was performed with a layer thickness of 160 μm, resulting in nine deposited layers with a theoretical width of 200 μm. The extrusion temperature was set at 190 °C, the build plate temperature at 45 °C, and the printing speed at 6 mm s^−1^.

**Figure 1:**
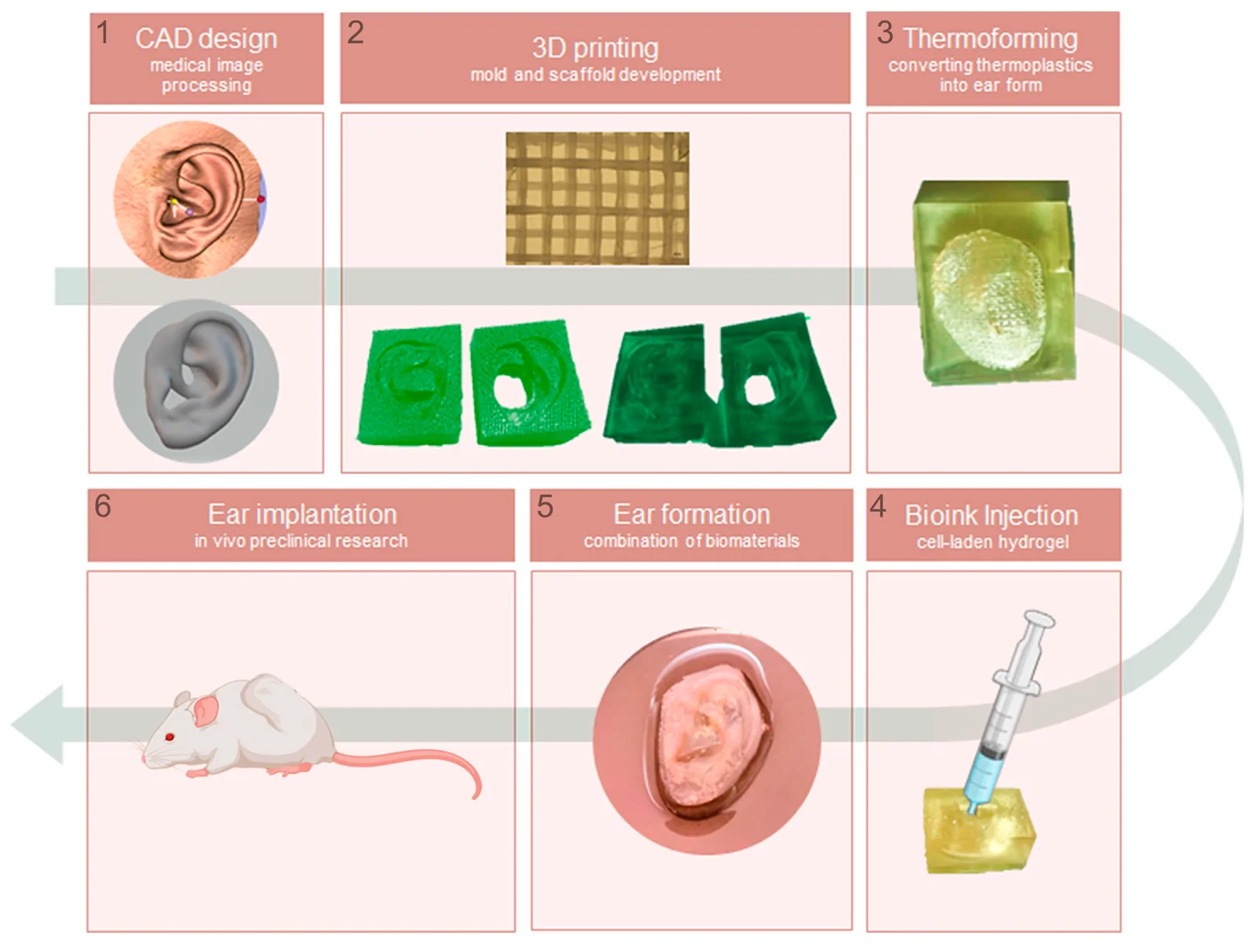
Schematic illustration of the steps to create the thermoformed ear-shaped scaffolds and implant them in vivo.

Hereafter, the 2.5 × 1.5 cm^2^ ear-shaped construct thermoformed scaffolds were fabricated by digitally modeling the ear of a healthy volunteer using a DICOM sequence derived from a high-resolution CT dataset acquired by computed tomography (Siemens Healthineers, Somaton force, Herlangen, Germany). The reconstructed three-dimensional geometry was converted into a physical model by additive manufacturing and subsequently employed as a mold for scaffold thermoforming. Flat PLA-PEG scaffolds (2.5 × 1.5 cm^2^) were then thermoformed at 70 °C for 10 min using the printed ear-shaped mold, enabling the generation of anatomically shaped auricular constructs (Figure 1).

The fabricated scaffolds, prior to the in vitro and in vivo assays, underwent a sequential 10 min immersion in 70% ethanol and subsequent washing with sterile phosphate-buffered saline (PBS) solution. The process was repeated three times to ensure effective cleanliness. Hereunder, the scaffolds were sterilized by exposure to ultraviolet (UV) irradiation for 30 min on each side.

### 2.2. Human Chondrocyte Isolation, Culture, and Expansion

Nasal septum and costal cartilage samples were obtained from patients undergoing rhino septoplasty and DIEP flap breast reconstruction, respectively, under informed consent (CEIC: 2019.100). Cartilage samples were minced and enzymatically digested in a combined solution of 10 mg/mL hyaluronidase (Sigma Aldrich, St. Louis, MA, USA) and 10 mg/mL type II collagenase (Worthington Biochemical, Lakewood, NJ, USA) in DMEM medium, at 37 °C in sustained soft agitation for 12 h (10 mL per gram of tissue). Subsequently, the enzymatic digestion was inactivated, and the samples were centrifuged at 300× *g* for 10 min. The total number of cells obtained from each patient was counted using the Cellometer Auto X4 (Nexcelom, Lawrence, MA, USA). Chondrocytes were expanded for a maximum of two passages, in DMEM supplemented with 10% fetal bovine serum (FBS), 1% Penicillin/Streptomycin, and 1% Insulin-Transferrin-Selenium (ITS) (Thermo Fisher Scientific, Waltham, MA, USA).

All the information regarding donor information and number of samples and animals (if necessary) used per experiment have been summarized in Table S1.

### 2.3. Assembly of the Cellularized Constructs

Expanded chondrocytes were detached with 0.05% trypsin (Sigma-Aldrich, St. Louis, MA, USA), centrifuged, and counted. Then, cells were resuspended at 18.75 × 10^6^ cells/mL in a 4% solution of sodium alginate low endotoxin AL10 (Alg, Kimica Corporation, Tokyo, Japan, Good Manufacturing Practice (GMP)-approved). Two different structures were tested in the in vivo experiments, a 1 × 1 cm^2^ squared scaffold, used in the maturation and mechanical testing, and the ear-shaped thermoformed scaffold. For the squared inserts, 80 μL of cellular suspension carrying 1.5 × 10^6^ of cells was injected on top of each scaffold and polymerized for 3 min in a 150 mM CaCl_2_ solution. For the full ear (Figure 1), the thermoformed PLA-PEG scaffold was placed in a three-dimensional mold and closed tightly, and the cell suspension in the sodium Alg was injected into the mold in small fractions until the contours were filled with a total of 1 mL with 18.75 × 10^6^ of cells. Then, it was sunk into the 150 mM of CaCl_2_ for 30 min and then carefully unmolded. Each implant was maintained in a DMEM medium enriched with 10% FBS and 1% Penicillin/Streptomycin. A suspension of Alg without cells was used as empty control in both in vivo experiments.

### 2.4. In Vitro Assays to Evaluate Different Cell Sources

The viability of cells was evaluated at 24 h and 7 days after polymerization using a live/dead cytotoxicity kit (Life Technologies, Carlsbad, CA, USA) following the manufacturer’s instructions. Briefly, 1 × 1 cm^2^ samples were incubated in Calcein AM solution (green) and ethidium homodimer-1 (red) solution for 30–45 min at 37 °C. After the incubation, samples were washed with D-PBS and the images were captured on a Leica Confocal Microscope (DMIL LED, Leica, Wetzlar, Germany).

To evaluate the capacity of each cell type to synthesize new matrix, a maturation assay was developed in vitro cultivating the cellularized scaffolds in DMEM supplemented with 10% fetal bovine serum (FBS), 1% Penicillin/Streptomycin, and 1% Insulin-Transferrin-Selenium (ITS) solution (Thermo Fisher Scientific, Waltham, MA, USA). They were histologically evaluated at 7 and 28 days.

### 2.5. Ectopic In Vivo Model

All procedures included in this study were approved by the University of Navarra Institutional Committee on Care and Use of Laboratory Animals (CEEA) and the Navarra Regional Government (CEEA #114-18). To determine the correct biocompatibility of the assembled materials and the proper time to obtain a suitable extracellular matrix (ECM) deposition, we developed an ectopic in vivo model in immunodeficient Rag2 mice (C;129S4-Rag2tm1.1Flv Il2rgtm1.1Flv/J) (Envigo, Indianopolis, IN, USA).

The animals were subjected to inhalation anesthesia, isoflurane (2%) (B. Braun Vet Care, Tuttlingen, Germany), and either four 1 × 1 cm^2^ structures (with 1.5 × 10^6^ nasal septum chondrocytes) or a single thermoformed auricular-shaped insert structure (with 18.75 × 10^6^ nasal septum chondrocytes) were implanted ectopically in a lower back subcutaneous pocket of Rag2 mice through a 2.5 cm skin incision. Acellular material, PLA-PEG scaffold with cell-free Alg hydrogel, was implanted as a control group. Histological, molecular biology, and biomechanical tests were performed at 3, 6, and 12 weeks post-implantation.

### 2.6. Histological and Immunofluorescence Analysis

For the histological analysis, mice were euthanized through CO_2_ saturation at the specific time points of the analysis. Those subcutaneous inserts for histological analyses were harvested and fixed in a 4% paraformaldehyde (PFA) solution for 24 h at room temperature. After fixation, the samples were dehydrated, embedded in paraffin, sectioned at 4 μm thickness, and stained for Hematoxylin & Eosin (H&E), Toluidine Blue (TB), and the Verhoeff’s stains. For the Verhoeff’s stain after deparaffinization and hydration, tissue sections were immersed in Bouin’s solution for 30 min and contrasted with Hematoxylin for 20 min.

For the immunofluorescence analysis, a sequential antigen unmasking was performed with 4 mg/mL of hyaluronidase (Sigma-Aldrich, St. Louis, MO, USA) in PBS, pH 5.5, for 15 min at 37 °C, followed by 4 mg/mL of pepsin (Sigma-Aldrich, St. Louis, MO, USA) in HCl 0.01 N for 30 min at 37 °C. The staining with type II collagen (1:2000, MP Biomedicals, Santa Ana, CA, USA, #631711) and type X collagen (1:100, Millipore, Burlington, MA, USA, #234196) were incubated in separate sections with primary antibodies overnight at 4 °C in a humidified chamber. Primary antibody was washed with PBS, and slides were incubated with 1:1000 dilution of Alexa Fluor^®^488 (Invitrogen, Carlsbad, CA, USA, #A11029/A11034) or Alexa Fluor^®^568 (Invitrogen, #A11004/A11011) secondary antibody for 1 h at room temperature. All preparations were counterstained with a 1:1 DAPI-Glycerol (Thermo Fisher Scientific, Waltham, MA, USA) solution and mounted in VECTASHIELD^®^ Antifade Mounting Medium (Vector Laboratories, Newark, CA, USA).

### 2.7. Bulk-RNA Library Preparation, Sequencing, Analysis, and Validation

The inserts to perform molecular analyses were harvested, washed in D-PBS, and placed in an RNAse-free Eppendorf in ice. RNA was extracted using TRIzol (Life Technologies, Carlsbad, CA, USA), following the manufacturer’s instructions. Briefly, the 1 × 1 cm^2^ squared inserts (for qPCR) or a small, randomized section of the cellularized ear-shaped scaffold (for bulk RNA-seq) were dissolved in TRIzol. After chloroform extraction, total RNA was purified from the aqueous solution using the RNeasy Plus Mini Kit (Qiagen, Hilden, Germany) following manufacturer instructions. A pellet containing 5 × 10^5^ NC before implantation was used as the control sample.

RNA quality was measured employing the High Sensitivity RNA ScreenTape kit (Agilent Technologies, Santa Clara, CA, USA) and following manufacturer instructions. Roughly 150 ng of total RNA per sample were used for transcriptomic interrogation of pelleted chondrocytes (*n* = 4) and the ear-shaped cellularized scaffold (*n* = 3) using Illumina Stranded Total RNA Prep Ligation with Ribo-Zero Plus (Illumina, San Diego, CA, USA), according to the manufacturer’s instructions. Libraries were quantified using Qubit dsDNA HS Assay Kit (Thermo Fisher, Waltham, MA, USA), and fragment size distribution was assessed by Agilent’s HS D1000 ScreenTape Assay (Agilent Technologies, Santa Clara, CA, USA). Sequencing was performed on an Illumina NextSeq 2000 platform using paired-end, dual-index sequencing (Read 1: 59 cycles; i7: 10 cycles; i5: 10 cycles Read 2: 59 cycles) at a depth of 50 million reads per sample.

Samples were demultiplexed using Illumina blc2fastq software (v2.2.0), reads trimmed, and quality filtered with Trim Galore (v0.6.10) and aligned to both the human (GRCh38) and mouse (mm39) reference genomes separately with STAR (V2.7.11b) using default parameters. Reads from BAM files were confidently assigned to either mouse or human with ngs_disambiguate (v1.0) using default parameters, filtering out ambiguous reads. For human, gene expression quantification was performed using the featureCounts function implemented in the R package Rsubread (v2.20.0), counting uniquely mapped reads with reverse strandness. The Ensembl v115 gene annotation was considered as a reference. Downstream analyses were conducted in R (v4.4.0). Lowly expressed genes were filtered out with the filterbyExpr function implemented in the edgeR package (v4.4.2), keeping only protein coding genes. Data normalization, transformation (considering variance stabilizing transformation), principal component analysis, and differential expression analyses were performed using the DESeq2 package (v1.46.0). The Wald test was used for the pairwise comparison, applying false discovery rate correction (FDR < 0.05) and considering an absolute shrunken log2 fold change (corrected with the lfcShrink function) above 1. Functional overrepresentation analysis (ORA) of the differentially expressed genes was performed using enrichR (v3.4) against the “GO_Biological_Process_2025” and “Reactome_Pathways_2024” databases. Pathway activity was inferred using the PROGENy (Pathway RespOnsive GENes for activity inference) resource implemented through decoupleR (v2.12.0) and including a curated collection of pathways and target genes with weights for interactions. Activity scores per sample and pathway were computed with run_mlm, considering the vst normalized counts.

For the qPCR validation, 1 μg of total retrotranscribed RNA was used with a qScriptTM Supermix (Quantabio, Beverly, MA, USA). The qPCR was performed in a Quantstudio 5 PCR system (Applied Biosystems, Foster City, CA, USA) using Taqman probes (Life Technologies, Carlsbad, CA, USA) for *Aggrecan (ACAN, Hs00153936_m1), Collagen Type II Alpha 1 Chain (COL2A1, Hs00264051_m1), Collagen Type I Alpha 1 Chain (COL1A1, Hs00164004_m1), Vascular Endothelial Growth Factor A (VEGFA, Hs00900055_m1), (PRG4, Hs00981633_m1), Collagen Type XI Alpha 1 Chain (COL11A1, Hs01097664_m1), SRY-box transcription factor 9 (SOX9, Hs00165814_m1), Parathyroid Hormone Like Hormone (PTHLH, Hs00174969_m1)*, and *Matrix Metalloproteinase 13 (MMP-13, Hs00942584_m1)*.

### 2.8. Biomechanical Analysis

The mechanical properties, including compression strength of the simplest scaffolds (1 × 1 cm^2^), were evaluated using a UniVert CellScale tensile test machine (CellScale Biomaterials Testing, Waterloo, ON, Canada). The analysis was carried out by using a 200 N load cell with an accuracy of ±0.5%, applying 30 mm diameter plates of stainless steel for all samples. Testing proceeded at a deformation rate of 25% per minute until reaching 50% deformation, with an initial manual preload of 1 N. Throughout the testing, samples were maintained in DMEM cellular medium at 37 °C to simulate physiological conditions.

### 2.9. Statistical Analysis

Graphical results are expressed as mean ± standard error of the mean. Statistical analysis was performed using GraphPad Prism 10.0 software (GraphPad Software Inc., San Diego, CA, USA). Gaussian distributions were analyzed with the Kolmogorov–Smirnov test. Single comparisons were analyzed by Student *t*-test or Mann Whitney U test, and multiple comparisons, with one-way ANOVA or Kruskal Wallis test followed by Sidàk’s or Dunn’s post hoc analysis, respectively. Significance was set at *p* < 0.05.

## 3. Results

### 3.1. Nasal Septum Chondrocytes Demonstrate Increased Capacity of New Matrix Generation In Vitro

**Figure 2:**
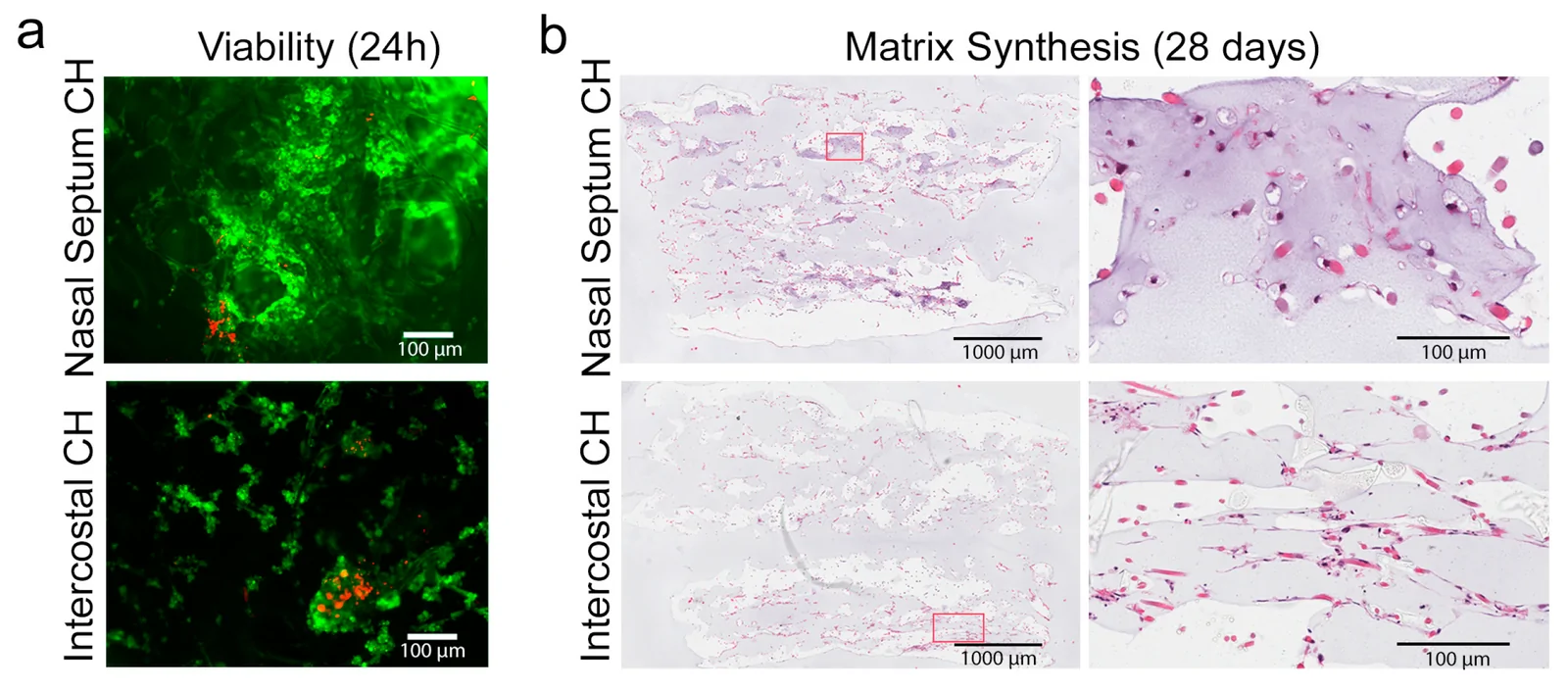
Viability and maturation in vitro tests of costal and nasal septum chondrocytes to select the most suitable cellular source. (**a**) Viability results after 24 h. Calcein AM solution (green, live) and ethidium homodimer-1 (red, dead). (**b**) Maturation test at 28 days. Representative Hematoxylin & Eosin images showing ECM deposition, with an overview on the left and a magnified close-up on the right (from the red box marked on the left images).

One of the critical points to create a whole ear is determining the most suitable cell source able of synthesizing the characteristic extracellular matrix (ECM) of this organ, maintaining the whole structure for a successful surgical treatment. Freshly expanded chondrocytes from two different sources, the nasal septum and costal cartilage, were embedded in alginate (Alg) and seeded into independent 1 × 1 cm^2^ squared inserts with double-offset geometry. This pattern was selected based on previous works published by our group [23]. Viability was tested at 24 h (Figure 2a), and their capacity of ECM deposition and maturation was evaluated at 28 days (Figure 2b). Both cell types showed equivalently high viability 24 h post-seeding, with most cells positive for Calcein staining (green), demonstrating that the assembly and polymerization processes did not negatively impact cell viability. However, only nasal septum chondrocytes displayed ECM deposition by HE staining within the alginate, both at 7 days (Figure S1) and 28 days (Figure 2b). These results confirmed nasal septum chondrocytes as the most suitable cellular source.

### 3.2. Simplified Engineered Inserts In Vivo Model Shows Improved Viscoelastic Properties with Nasal Chondrocytes

Mimicking the mechanical properties of natural tissue is an essential feature for any bioengineered 3D construct. However, the complex intrinsic shape of the auricular pavilion presents significant regional variations that complicate the evaluation of specific material characteristics with or without cells [24]. Therefore, simplified structures of PLA-PEG + Alg (1 × 1 cm^2^ squared inserts) were evaluated in an ectopic in vivo model to define the benefits of including NC in an engineered auricular pavilion (Figure S2a). These inserts were maintained in vivo for 12 weeks to compare variations in mechanical properties between PLA-PEG + Alg without cells (Empty) or cellularized constructs (Cell-laden), considering the hybrid composition of the developed inserts. The mechanical behavior of the structures was evaluated under simulated physiological conditions.

**Figure 3:**
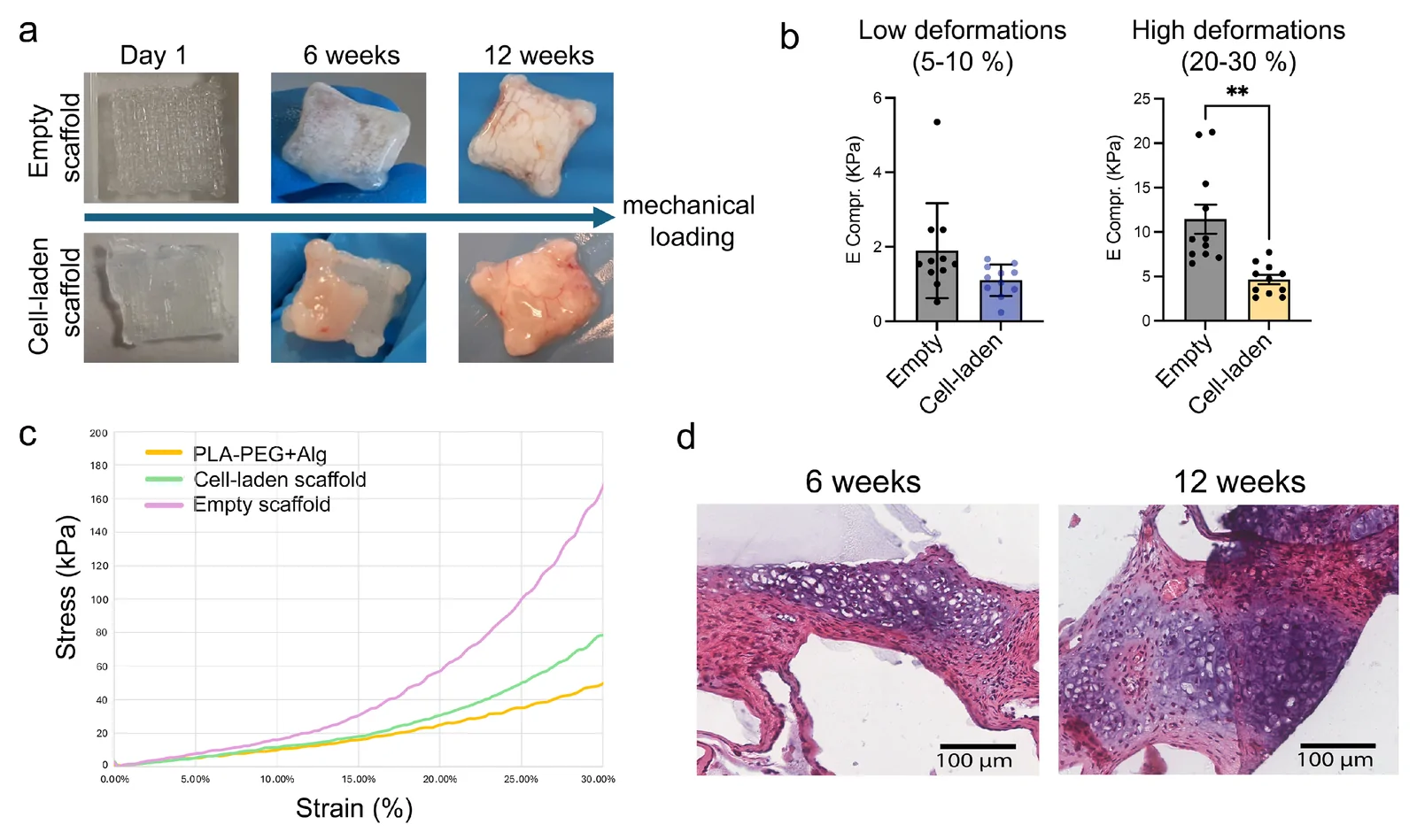
Mechanical and histological analyses of simplified cellularized scaffolds after ectopic implantation. (**a**) Macroscopic appearance of the constructs at 6 and 12 weeks post-implantation before the mechanical test. (**b**,**c**) Mechanical results at low and high deformations in empty (without cells) and cell-laden scaffolds at 12 weeks, *p* < 0.01 (**), and (**d**) Representative Hematoxylin & Eosin images showing chondrogenic areas in the cell-laden scaffolds at 6 and 12 weeks post-implantation.

The macroscopic view of the inserts after ectopic in vivo implantation (Figure 3a) evidenced differences between the cellularized inserts, which had a translucent appearance, and the empty control samples, which displayed a whitish appearance similar to calcified tissues. A thin layer of vascularized tissue covered the engineered cellularized implants and showed a soft appearance.

To understand the relevance of incorporating cells into the structure, the variation of the mechanical behavior between the polymer PLA-PEG and PLA-PEG + Alg was first determined to isolate the viscoelastic behavior of the empty material. The Young’s modulus decreased with the presence of Alg (Figure S2b,c). The incorporation of chondrocytes in the constructs resulted in samples exhibiting a sustained mechanical behavior with lower Young’s modulus value, indicative of lower resistance to elastic deformation. These differences were significant only at higher deformations (Figure 3b,c). Empty samples showed an exponential increase of Young’s modulus typical of stiff materials (Figure 3b,c).

The evaluation of the inserts was completed by histological analysis at equivalent time points (Figure 3d and Figure S2d). H&E staining revealed chondrogenic areas in the engineered inserts at 3, 6, and 12 weeks. However, a higher number of chondrogenic areas was detected at 12 weeks, which supported selecting this time point for subsequent experiments. These experiments indicate that the maturation advanced directly in vivo within the cellularized implants, improving the biomechanical characteristics of the engineered tissue.

### 3.3. The Refined Ear-Shaped Cellularized Scaffolds in the Ectopic In Vivo Shows That the New Synthetized Tissue Derived Exclusively from Human Cells

**Figure 4:**
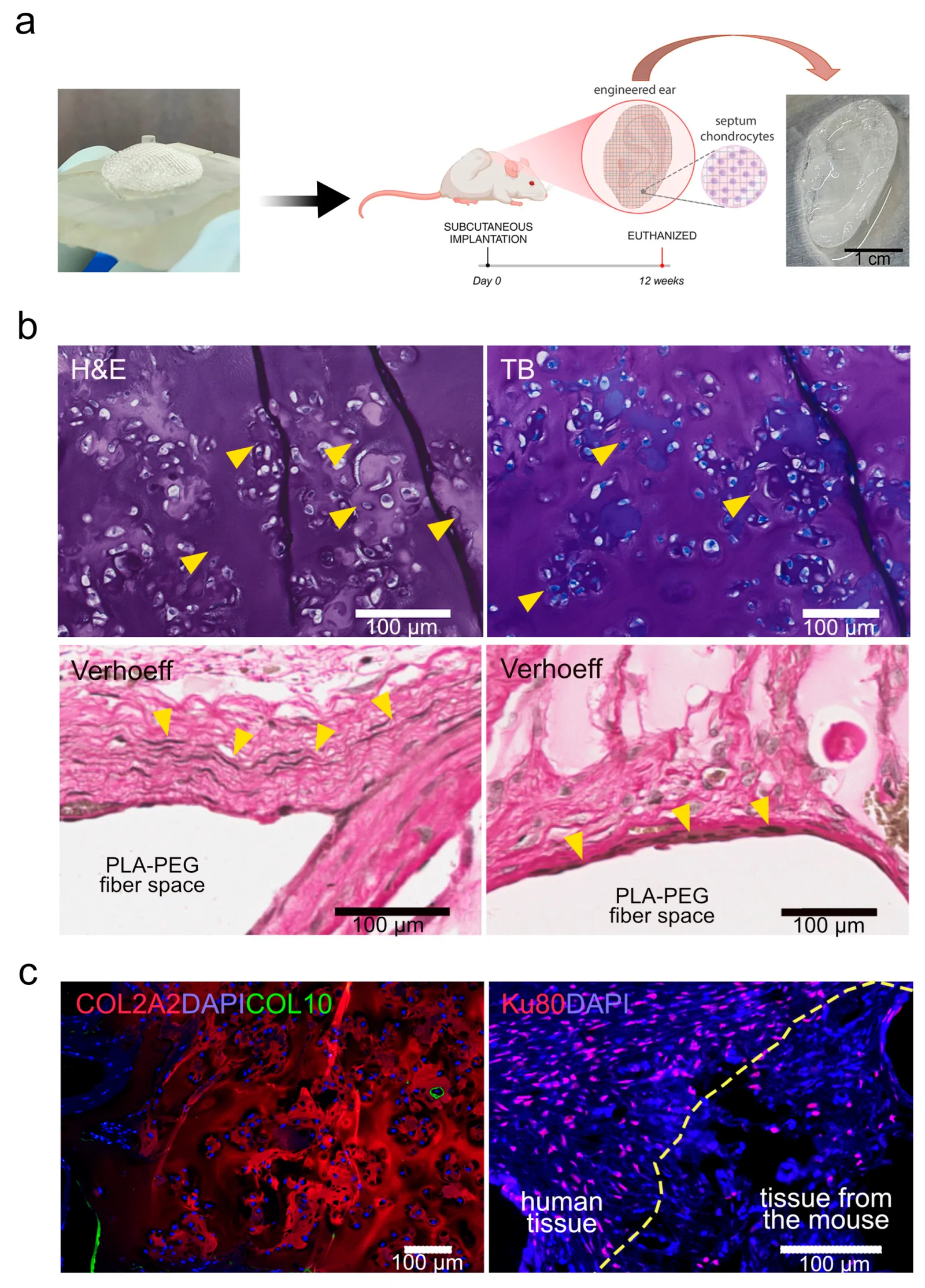
Ear-shaped cellularized scaffolds show proper synthesis of new tissue, mainly derived from the human cells in vivo. (**a**) Schematic illustration of the ectopic in vivo model including the timeline and a photograph of the cellularized ear before implantation. (**b**) In the upper panels, histological analysis of the ear after 12 weeks of implantation showed a proper structure of matrix and chondrogenic areas (yellow arrows) with Hematoxylin & Eosin (H&E) staining and the presence of glycosaminoglycans (GAG) with the Toluidine Blue (TB) staining (yellow arrows). In the bottom panels, Verhoeff’s staining revealed mature elastic fiber deposition (left, yellow arrows) and increased staining intensity on the cells close to the PLA-PEG fibers. (**c**) Immunofluorescence analysis of the implants showed the presence of collagen type II deposition and absence of hypertrophy (negative for collagen type X). Cellularized scaffolds are positive for Ku80, specific to human cells, surrounded in the exterior areas for a fibrotic mouse tissue (marked by a yellow dashed line for clarity).

Once the appropriate cell source and the optimal maturation time in the ectopic model were established, we fabricated the ear-shaped thermoformed scaffolds and implanted them in vivo. Scaffolds were fabricated to resemble the ear of a <1 year-old healthy volunteer (see Figure 1 for detailed steps) and seeded at a concentration of 18.75 × 10^6^ cells/mL to maintain the same cell-to-volume proportion as in the previous scaffolds. The engineered external ear was evaluated in the ectopic model at 12 weeks post-implantation (Figure 4 and Figure S3).

Histological analysis using H&E and TB showed numerous regions of chondrogenic areas and new ECM deposition throughout all regions of the inserts, with a proper presence of glycosaminoglycans (Figure 4b). The Verhoeff’s stain analysis revealed localized elastic fiber deposition in a small area of one out of three thermoformed samples (Figure 4b, bottom panel). In those samples, the fibers were observed at the periphery of PLA-PEG fibers accompanied by an increased staining intensity on the cell nuclei.

Specific immunofluorescence analysis for Type II Collagen (hyaline-like cartilage) and Type X Collagen (hypertrophic-like cartilage) indicated mature hyaline cartilage with a high presence of Collagen type II and scarce presence of Collagen type X in the newly synthesized matrix (Figure 4c). Moreover, to verify whether human cells were responsible for synthesizing the new matrix, immunofluorescent staining for human Ku80 was performed. All regions with a newly synthesized matrix where collagen type II was detected were also positive for the Ku80 marker. This confirms that the cells participating in new tissue synthesis were predominantly of human origin.

These results verify the synthesis of a chondrogenic ECM with slight presence of elastic fibers.

### 3.4. Transcriptomic Profiling Indicates a Robust Chondrogenic Maturation of the Synthesized Tissue

To evaluate the maturation state of the cells in the ear-shaped thermoformed scaffolds after 12 weeks in vivo, we performed bulk RNA-seq. For that, ear-shaped thermoformed scaffolds (*n* = 3) were sectioned in small pieces to evaluate comparable areas across ears against pre-implantation NC (*n* = 4).

**Figure 5:**
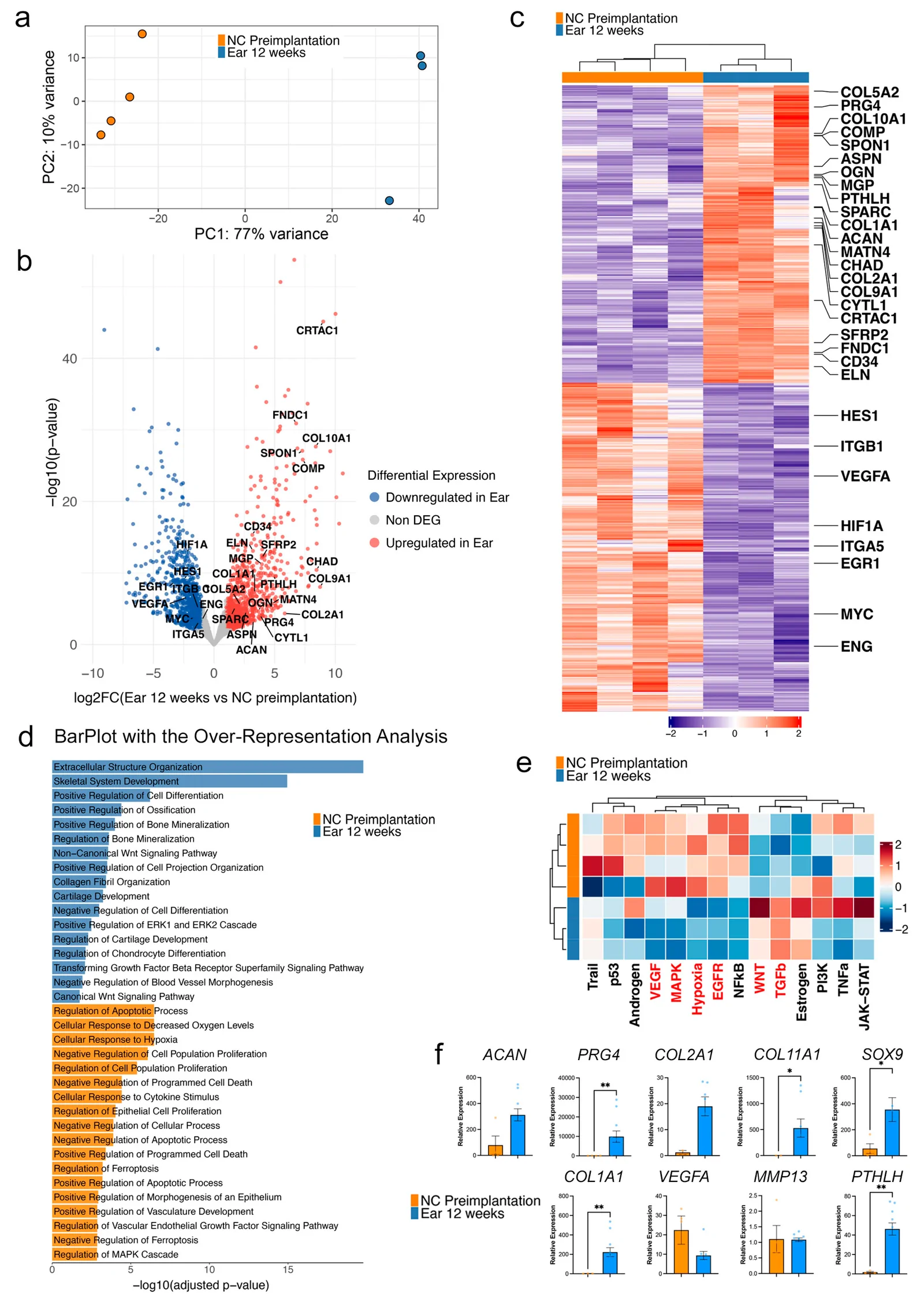
Transcriptional analysis of the ear-shaped thermoformed scaffolds (in blue) compared to pre-implantation NC (in orange). (**a**) Principal components analysis, (**b**) volcano plots, and (**c**) heatmap of differentially expressed genes (only genes of interest are labeled). Functional analysis from the bulk RNA-seq data through (**d**) overrepresentation analysis considering the Gene Ontology (GO) Biological Processes database and (**e**) curated pathway scores from PROGENy. (**f**) Validation of the analyzed bulk RNA-seq data by qPCR, *p* < 0.05 (*) and *p* < 0.01 (**).

Following quality control and data pre-processing, we compared the initial NC pellet with the ear-shaped thermoformed scaffold conditions 12 weeks after implantation. Principal component analysis (PCA) showed that PC1 accounted for most of the variance (77%) and clearly separated both groups, pointing to consistent differences between them (Figure 5a). A total of 1780 differentially expressed genes (DEG) were identified between the two groups considering an absolute log2 fold change of 1. This included 846 upregulated and 934 downregulated protein coding genes (Figure 5b,c). The results showed a clear upregulation of genes related to chondrogenesis or matrix synthesis in the ear-shaped scaffolds. We observed increased expression of different collagen genes, such as *Collagen Type II Alpha 1 Chain (COL2A1)*, *Collagen Type V Alpha 2 Chain (COL5A2)*, *Cartilage Oligomeric Matrix Protein (COMP)*, and *Matrilin 4 (MATN4)*, all encoding structural protein components of the matrix, as well as genes involved in the control of collagen trimerization, such as *Asporin* (*ASPN*). In addition, we found an increased expression of *Aggrecan (ACAN)* and *Proteoglycan 4 (PRG4)*, which code for glycosaminoglycans, key components of the cartilaginous extracellular matrix. Another distinctive extracellular matrix component of the native ear cartilage is the presence of elastic fibers. We observed transcriptional upregulation of *Elastin (ELN)*, the major structural molecule that forms one of the two components of elastic fibers in the extracellular matrix (Figure 5b). Upregulation was also observed for genes involved in cell adhesion and chondrocyte growth/differentiation, such as *Spondin 1 (SPON1)*, *Chondroadherin (CHAD)*, and *Secreted Frizzled Related Protein 2 (SFRP2)*. Importantly, the expression of genes associated with a mature cartilaginous matrix was not accompanied by the expression of genes related to hypertrophy and calcification processes, suggesting a maintenance of maturity without generating a calcification process in the tissue. Osteogenic-related genes were also found to be differentially expressed, with an increase in genes like *Cartilage Acidic Protein 1 (CRTAC1)* and *Osteoglycin (OGN)*, indicative of osteoblast differentiation, and *Matrix Gla Protein* (*MGP*), an inhibitor of ectopic tissue calcification.

This correlates with the results from an overrepresentation analysis, describing an upregulation of biological processes related to extracellular matrix organization and cell differentiation in the ear-shaped thermoformed inserts (Figure 5d). Using PROGENy (Pathway RespOnsive GENes for activity inference) for pathway activity inference, the ear-shaped inserts showed higher activity scores mainly for TGFβ and WNT, related to chondrogenesis and osteogenesis, while there was a decreased activity for pathways such as Hypoxia, VEGF (vascularization), NF-κB (inflammation or catabolic processes), and EGFR (cell proliferation) (Figure 5e), in line with the differential processes and genes. An overrepresentation analysis considering the Reactome database retrieved differential processes related to elastic fiber formation due to an increased expression of nine additional genes, besides *ELN*, related to those pathways in our engineered samples (Table S2). These include *MFAP5 (Microfibril-Associated Protein 5)*, *MFAP4 (Microfibril-Associated Protein 4)*, *ITGB5 (Integrin Beta-5)*, *TGFB3 (Transforming Growth Factor Beta 3)*, *EMILIN3 (Elastin Microfibril Interfacer 3)*, *EMILIN2 (Elastin Microfibril Interfacer 2)*, *LTBP2 (Latent Transforming Growth Factor Beta Binding Protein 2)*, *FBLN2 (Fibulin 2)*, and *BMP7 (Bone Morphogenetic Protein 7)* (Figure S4).

Validation by qPCR confirmed an upregulation trend in mesenchymal differentiation, evidenced by elevated expression of *parathyroid hormone like hormone (PTHLH)* and *SRY-Box transcription factor 9 (SOX9)*, along with matrix component synthesis genes such as *Aggrecan (ACAN), Proteoglycan 4 (PRG4), Collagen type II alpha 1 chain (COL2A1)*, and *Collagen type XI alpha 1 chain (COL11A1*). Although *Collagen type 1 alpha 1 chain* (*COL1A1*) expression showed significant upregulation compared with the pre-implanted cellular pellet, immunofluorescence analysis showed barely detectable protein levels (Figure 4b). Notably, there was a decreased expression of the *Vascular Endothelial Growth Factor* (*VEGFA*) on the inserts, indicating reduced angiogenic demand in the newly formed tissue, in alignment with the PROGENy pathway analysis (Figure 5f).

Together, these results confirm a successful synthesis of new cartilage matrix and proper native-like tissue maturation without inducing calcification. Therefore, the materials and cells used in these structures offer an appropriate approach for developing auricular substitutes.

## 4. Discussion

Tissue Engineering has led to a paradigm shift in regenerative and reparative medicine. Although the first engineered tissues for auricular reconstruction were produced in the early 90s, three decades later, the treatment for traumatic or congenital ear absence still relies predominantly on autologous costal cartilage implantation. Autologous treatment offers unmatched benefits in terms of proper preservation of the ear shape over time and absence of rejection. However, it also presents numerous disadvantages: it involves an aggressive mutilating surgery that causes donor site morbidity and pain, is highly dependent on surgical expertise, often demands multiple surgical stages, and may carry other adverse complications such as pneumothorax or chest thoracic wall deformity [25].

Despite recent advances in Tissue Engineering, only one polyethylene synthetic material, MedPore^®^ (Stryker, Kalamazoo, MI, USA), has been approved for clinic ear reconstruction. Nevertheless, clinical reports indicate that complications such as extrusion, poor esthetic results, and high infection incidence have limited its widespread adoption [5,26]. Recent development in new biocompatible materials and bioprinting technology have enabled the fabrication of whole tissues and organs, including the human auricle [27]. Among the key advantages of these fabrication approaches is the ability to accurately mimic the shape and size of the contralateral ear of the patient. The structure can be generated in a fast and efficient manner, with the option of testing complex combinations of materials and cells. However, seamless clinical translation remains far from being fully achieved.

In this work, we have developed a cellularized engineered ear construct. The construct combines a thermoformed PLA-PEG scaffold, which provides the necessary mechanical support and shape fidelity to replicate the intricate auricular architecture, with a clinical-grade sodium alginate hydrogel encapsulating human chondrocytes. This hybrid strategy addresses the limitation of alginate hydrogels, which, despite their excellent biocompatibility, exhibit insufficient mechanical strength and structural stability when used alone, limiting their ability to maintain complex 3D shapes in cartilage tissue engineering [28,29,30]. We have successfully defined NC as the most appropriate cell source in combination with these materials over intercostal autologous chondrocytes. Although autologous chondrocytes have been considered the ideal conceptual approach to cell therapies in patients, recent single-cell transcriptomic analyses have uncovered significant molecular alterations when comparing chondrocytes from microtia patients and healthy donors [20,31]. Microtia-derived chondrocytes exhibit a reduced presence of chondral progenitor cells and mitochondrial dysfunction with increased levels of reactive oxygen species (ROS). Altogether, this would hamper chondrocyte expansion and differentiation [32] and proper ECM synthesis, compromising the therapeutic success of these therapies. In this work, we have evaluated two clinically relevant cell sources: costal and nasal septum chondrocytes. The in vitro analysis and the simplified material implanted ectopically in vivo demonstrated that NC had superior capacity to synthesize new ECM, which, together with their great expansion ratio, made them more appropriate for these inserts.

When evaluating the full-scale ear constructs in vivo, histology and immunofluorescence analysis at 12 weeks confirmed a robust synthesis of ECM through the presence of chondrogenic areas positive for collagen type II and proteoglycans despite a mid-loss of the intricate anatomical definition over time. Crucially, the newly synthesized tissue showed a positive immunofluorescence for human Ku80, indicating a prevalent participation of the human cells in the new synthetized matrix.

Transcriptomic interrogation verified the histological findings. We observed a significant upregulation of a plethora of genes related to ECM synthesis, such as *COL2A1*, *COL5A3*, *COMP*, *MATN4*, *ACAN*, and *PRG4*. At 12 weeks post-implantation, prevailing processes were related to matrix deposition and organization, as well as cell proliferation. Although a slight increase in the expression of *COL1A1* was detected in the inserts, indicating a modest fibrotic expression activation in the NC, the relative values are low and the histological analysis did not show any COL1A1 deposition, likely reflecting a local host reaction [33]. Native auricular cartilage possesses a unique network of elastic fibers that confers the mechanical flexibility of this tissue. In our molecular analysis, *ELN (Elastin*), part of the two components of elastic fibers in the extracellular matrix, was significantly overexpressed in the thermoformed scaffold. Although *FBN1 (Fibrillin-1)*, a structural glycoprotein that provides tensile strength and elastic fiber scaffolding, was not differentially expressed in the 12-week samples, there was a significant overrepresentation of Reactome pathways related to elastic fibers due to increased expression of nine associated genes, including *MFAP5*, *MFAP4*, *ITGB5*, *TGFB3*, *EMILIN3*, *EMILIN2*, *LTBP2*, *FBLN2*, and *BMP7*. These results indicate that transcriptional regulation alone is insufficient to drive elastic fiber formation, although the process is activated. These combined results indicate that transcriptional machinery for elastogenesis is activated in our constructs. However, the transcriptional upregulation alone is insufficient to drive elastic fiber formation. It is likely linked to a proper post-transcriptional regulation, changes in the extracellular assembly of Tropoelastin and fibrillin molecules, and appropriate biomechanical microenvironment that was not reached in our in vivo model.

It is worthy to highlight a marked downregulation of *VEGF* signaling in the engineered cartilage tissue. Although *VEGF* is necessary during cartilage development, postnatal differentiation of articular chondrocytes, and ossification of bones in joint regions [34], mature healthy elastic and articular cartilage is strictly avascular. An increase in *VEGF* expression in adult cartilage is associated with pathological processes such as osteoarthritis (OA), usually correlating with disease severity [35]. In clinical ear reconstruction procedures, the initial vascularization for insert survival is provided by local flaps that replace the natural vascular nutrition function performed by posterior auricular arteries (PAA) and some perforating vessels [36]. Therefore, the minimal presence of angiogenic pathways reinforces the idea of a favorable chondrogenesis phenotype of the cellularized thermoformed-ear suitable for clinical translation.

Biomechanical testing of simplified constructs revealed that at low deformations (5–10%), load is supported by the Alg hydrogel, whereas at higher deformation (20–30%) is borne by the PLA-PEG structure. Cellularized implants exhibited a significantly improved viscoelastic behavior, probably as a consequence of the new matrix deposition, without requiring other external factors, such as a pre-incubation period, biomechanical stimulation, or maturation complements. [37]. Even if we are still far from the natural mechanical behavior of a real ear, it is a great advantage to facilitate clinical translation. On the other hand, regarding degradation, while PLA-PEG thermoformed scaffolds remained intact after 12 weeks, alginate hydrogel showed signs of degradation resulting in loss of the fine auricular details.

Current literature highlights strategies to overcome the limitations of conventional auricular reconstruction focusing on multifunctional scaffolds that simultaneously address anatomical fidelity, mechanical stability, cellular activity, and tissue maturation. For instance, the incorporation of bacterial nanocellulose (BNC) into GelMA hydrogels significantly improves the mechanical strength, printability, cell migration, and deposition of ECM in vivo [38]. However, BNC is a non-degradable material and cannot be replaced by native tissue over time. Another innovative strategy, developed in the work of Hua et al. 2026 [39], proposes a transition from passive synthetic scaffolds toward patient-specific, cell-instructive, and biologically active multimaterial constructs. In this work, a double-network ElaMA/GelMA provides enhanced mechanical support, with a high resistance to tissue contraction and improved pore architecture. These materials incorporate platelet-derived extracellular vesicles (PEVs) to favor cartilage matrix deposition and immunomodulate the macrophage polarization, aiding the integrity of the scaffold during regeneration. Although current biomaterial scaffolds have developed notorious synthesis of ECM [40,41,42], improved anatomical fidelity, mechanical stability, and cellular activity, the multimaterial constructs increase the issues for a successful regulatory approach for clinical translation.

In contrast, there are alternative designs focused on understanding the natural behavior and development of native tissues rather than increasing the complexity of the scaffold. Huang et al. [43] introduced a new architecture that allows a mechanically resilient material to preserve auricular shape without incorporating different materials. In line with this idea, our strategy prioritizes elucidating the processes that allow the development of a complete mature cartilage. The idea of combining proper mechanical and biological development processes able to mimic the natural cues for tissue development with simplified materials, proper human cell sources, and Good Manufacturing Practice (GMP)-approved materials may facilitate compliance with the restrictive rules for clinical translation. Besides, an improved evaluation strategy for follow-up of the mechanical performance of ear cartilage scaffolds over time, such as a fractal-based method [44], would allow us to attain improved results.

Addressing these biomaterial–cell interactions and a proper technology adaptation for clinical purposes is essential to produce durable, clinically relevant engineered ears that closely mimic native auricular tissue.

## 5. Conclusions

In summary, this study presents an optimized manufacturing workflow for ear reconstruction combining human NC and clinically relevant translational materials. We identified septum chondrocytes as the most convenient cell source for ear reconstruction, integrated in a reinforcement of thermoformed PLA-PEG scaffold and GMP sodium alginate hydrogel. The resulting constructs demonstrated increased viscoelasticity properties and new ECM synthesis with appropriate maturation in vivo, confirmed by NGS and histological analysis, reinforcing the future of these engineered scaffolds as possible alternatives for traumatic or congenital ear loss. Nevertheless, certain limitations remain to be addressed prior to clinical translation. In our system, histological examination revealed localized areas resembling immature cartilage with fibrocartilaginous appearance, even when the transcriptional data showed a predominant appropriate maturation of the tissue. Future studies focusing on understanding the precise temporal remodeling dynamics in cartilage would determine the key factors to improve and accelerate the new tissue maturation. Besides, matching the hydrogel degradation rate with the new matrix synthesis will be essential to preserve fine anatomical details over extended periods. Nevertheless, our platform represents a promising alternative for anotia treatments to improve the well-being of patients.

## Data Availability

The bulk RNA-seq data generated in this study have been deposited in the NCBI GEO Expression Omnibus (GEO) repository (https://www.ncbi.nlm.nih.gov/geo/, access on 12 August 2026) under accession number GSE335549.

## Supplementary Materials

The following supporting information can be downloaded at: https://www.mdpi.com/article/10.3390/jfb17090453/s1, Table S1: Summary of cell’s donors, characteristics, number of samples and experiments used in this work. Table S2: Pathways upregulated in Ear at 12 weeks compared to NC preimplantation for the Reactome_Pathways_2024 databases. Highlighted the pathways related to elastic fibers. Figure S1: General and detailed view of the 1 × 1 cm^2^ squared inserts after 7 days of maturation in vitro comparing the two diferent cell sources; Figure S2: Scheme of the ectopic in vivo with the simplified cellular engineered inserts to perform the mechanical analysis (a). Mechanical results representation at low_and high deformations of the dfferences between PLA-PEG scaffolds without and with the Alginate in the structure to determine the viscoelastic behavior of the inserts without cells (b,c). Histological analyses through hematoxylin and eosin staining at 3 weeks (d), and the cellular-engineered squared inserts placed in the UniVert CellScale tensile plates at the moment to perform the mechanical analysis (e); Figure S3: General and detailed view of the histologic, through Hematoxylin-Eosin (H&E) staining and Toluidin Blue (TB), and immnunofluorescence analysis, through the COL2 (red) and_COL10 (green) with DAPI (blue, for the nucleous) and the_Ku80, positive for human cells (red), of the ear-shaped thermoformed scaffolds 12 weeks after implantation (yellow dashed line to define the limit between mouse and human tissues); Figure S4: Bar graphs showing the overexpression of genes related to elastic fibers formation at 12 weeks after implantation.

## Abbreviations

The following abbreviations are used in this manuscript:

Alg	Sodium Alginate low endotoxin
*ACAN*	*Aggrecan*
*COMP*	*Cartilage Oligomeric Matrix Protein*
*COL2A1*	*Collagen Type II Alpha 1 Chain*
ECM	Extracellular Matrix
*ELN*	*Elastin*
H&E	Hematoxylin and Eosin staining
*MATN4*	*Matrilin 4*
NC	Nasal Chondrocytes
PLA-PEG	Poly(lactide-b-ethylene glycol) copolymer
*PRG4*	*Proteoglycan 4*
qPCR	Quantitative Real Time PCR
TB	Toluidine Blue

## References

[1] Butler D.F., Gion G.G., Rapini R.P. (2000). Silicone auricular prosthesis. J. Am. Acad. Dermatol..

[2] Gao S., Nie T., Lin Y., Jiang L., Wang L., Wu J., Jiao Y. (2024). 3D printing tissue-engineered scaffolds for auricular reconstruction. Mater. Today Bio.

[3] Baluch N., Nagata S., Park C., Wilkes G.H., Reinisch J., Kasrai L., Fisher D. (2014). Auricular reconstruction for microtia: A review of available methods. Plast. Surg..

[4] Federspil P.A. (2010). Auricular Prostheses.

[5] Ma Y., Lloyd M.S. (2022). Systematic Review of Medpor Versus Autologous Ear Reconstruction. J. Craniofac. Surg..

[6] Su-Genyk P., Quatela O., Quatela V. (2024). Our Evolution of Approaches to Microtia Reconstruction. Facial Plast. Surg. Clin..

[7] Kim M., Kim Y.J., Kim Y.S., Roh T.S., Lee E.-J., Shim J.-H., Kang E.H., Kim M.J., Yun I.S. (2024). One-Year Results of Ear Reconstruction with 3D Printed Implants. Yonsei Med. J..

[8] Camison L., Lisk R., Soldanska M. (2025). Microtia. Clin. Plast. Surg..

[9] Mussi E., Furferi R., Volpe Y., Facchini F., McGreevy K.S., Uccheddu F. (2019). Ear Reconstruction Simulation: From Handcrafting to 3D Printing. Bioengineering.

[10] Aigner J., Bujía J., Hutzler P., Kastenbauer E. (1997). Distribution and viability of cultured human chondrocytes in a three-dimensional matrix as assessed by confocal laser scan microscopy. In Vitro Cell. Dev. Biol. Anim..

[11] Sittinger M., Bujia J., Rotter N., Reitzel D., Minuth W.W., Burmester G.R. (1996). Tissue engineering and autologous transplant formation: Practical approaches with resorbable biomaterials and new cell culture techniques. Biomaterials.

[12] Isogai N., Asamura S., Higashi T., Ikada Y., Morita S., Hillyer J., Jacquet R., Landis W.J. (2004). Tissue engineering of an auricular cartilage model utilizing cultured chondrocyte-poly(L-lactide-epsilon-caprolactone) scaffolds. Tissue Eng..

[13] Kusuhara H., Isogai N., Enjo M., Otani H., Ikada Y., Jacquet R., Lowder E., Landis W.J. (2009). Tissue engineering a model for the human ear: Assessment of size, shape, morphology, and gene expression following seeding of different chondrocytes. Wound Repair Regen..

[14] Vernice N.A., Dong X., Matavosian A.A., Corpuz G.S., Shin J., Bonassar L.J., Spector J.A. (2024). Bioengineering Full-scale auricles using 3D-printed external scaffolds and decellularized cartilage xenograft. Acta Biomater..

[15] Kozusko S.D., Konofaos P., Wallace R.D. (2020). The History of Alloplastic Ear Reconstruction for Microtia. Ann. Plast. Surg..

[16] Yamada A., Chwa E.S., Boctor M.J. (2024). Update on Total Auricular Construction. Plast. Reconstr. Surg..

[17] Sun H., Mei L., Song C., Cui X., Wang P. (2006). The in vivo degradation, absorption and excretion of PCL-based implant. Biomaterials.

[18] Höglund A., Hakkarainen M., Albertsson A.C. (2007). Degradation Profile of Poly(ϵ-caprolactone)–the Influence of Macroscopic and Macromolecular Biomaterial Design. J. Macromol. Sci. Part A Pure Appl. Chem..

[19] Christen M.O., Vercesi F. (2020). Polycaprolactone: How a Well-Known and Futuristic Polymer Has Become an Innovative Collagen-Stimulator in Esthetics. Clin. Cosmet. Investig. Dermatol..

[20] Li X., Li D., Zhang R. (2025). Single-Cell RNA sequencing reveals mitochondrial dysfunction in microtia chondrocytes. Sci. Rep..

[21] Otto I.A., Levato R., Webb W.R., Khan I.M., Breugem C.C., Malda J. (2018). Progenitor cells in auricular cartilage demonstrate cartilage-forming capacity in 3D hydrogel culture. Eur. Cell. Mater..

[22] Hanci D., Üstün O., Yilmazer A.B., Göker A.E., Kumral T.L., Uyar Y. (2020). Costal Cartilage and Costal Perichondrium Sandwich Graft in Septal Perforation Repair. J. Craniofac. Surg..

[23] Urtaza U., Guaresti O., Gorroñogoitia I., Zubiarrain-Laserna A., Muiños-López E., Granero-Moltó F., de Espinosa J.L., López-Martinez T., Mazo M., Prósper F. (2022). 3D printed bioresorbable scaffolds for articular cartilage tissue engineering: A comparative study between neat polycaprolactone (PCL) and poly(lactide-b-ethylene glycol) (PLA-PEG) block copolymer. Biomed. Mater..

[24] Buyuklu F., Cakmak M.P., Cakmak B.A., Durukan E., Hizal E. (2025). Age and Gender Related Variability in the Strength of Auricular Cartilage in Living Humans. Aesthetic Plast. Surg..

[25] Cao Y., Vacanti J.P., Paige K.T., Upton J., Vacanti C.A. (1997). Transplantation of chondrocytes utilizing a polymer-cell construct to produce tissue-engineered cartilage in the shape of a human ear. Plast. Reconstr. Surg..

[26] Ali K., Trost J.G., Truong T.A., Harshbarger R.J. (2017). Total Ear Reconstruction Using Porous Polyethylene. Semin. Plast. Surg..

[27] Huang Y., Zhao H., Wang Y., Bi S., Zhou K., Li H., Zhou C., Wang Y., Wu W., Peng B. (2023). The application and progress of tissue engineering and biomaterial scaffolds for total auricular reconstruction in microtia. Front. Bioeng. Biotechnol..

[28] Shi W., Chen S., Liu B., Omidian H., Dey Chowdhury S., Wilson R.L. (2024). Advancements and Challenges in Hydrogel Engineering for Regenerative Medicine. Gels.

[29] Ansari M., Darvishi A., Sabzevari A. (2024). A review of advanced hydrogels for cartilage tissue engineering. Front. Bioeng. Biotechnol..

[30] Javan Almasi M., Xiong D. (2024). Recent Progress in Bionic Hydrogels for Articular Cartilage: Tribological and Mechanical Characteristics. J. Bionic Eng..

[31] Ma J., Zhang Y., Yan Z., Wu P., Li C., Yang R., Lu X., Chen X., He A., Fu Y. (2022). Single-cell transcriptomics reveals pathogenic dysregulation of previously unrecognised chondral stem/progenitor cells in children with microtia. Clin. Transl. Med..

[32] Liu W., Wu Y., Ma R., Zhu X., Wang R., He L., Shu M. (2024). Multi-omics analysis of a case of congenital microtia reveals aldob and oxidative stress associated with microtia etiology. Orphanet J. Rare Dis..

[33] Mosala Nezhad Z., Poncelet A., Fervaille C., Gianello P. (2019). Comparing the Host Reaction to CorMatrix and Different Cardiac Patch Materials Implanted Subcutaneously in Growing Pigs. Thorac. Cardiovasc. Surg..

[34] Nagao M., Hamilton J.L., Kc R., Berendsen A.D., Duan X., Cheong C.W., Li X., Im H.-J., Olsen B.R. (2017). Vascular Endothelial Growth Factor in Cartilage Development and Osteoarthritis. Sci. Rep..

[35] Ma K., Pham T., Wang J., O-Sullivan I.S., DiCamillo A., Du S., Mwale F., Farooqui Z., Votta-Velis G., Bruce B. (2024). Nanoparticle-based inhibition of vascular endothelial growth factor receptors alleviates osteoarthritis pain and cartilage damage. Sci. Adv..

[36] Reinisch J.F., Tahiri Y. (2019). Modern Microtia Reconstruction: Art, Science, and New Clinical Techniques.

[37] Griffin M.F., Premakumar Y., Seifalian A.M., Szarko M., Butler P.E.M. (2016). Biomechanical Characterisation of the Human Auricular Cartilages; Implications for Tissue Engineering. Ann. Biomed. Eng..

[38] Zeng J., Jia L., Wang D., Chen Z., Liu W., Yang Q., Liu X., Jiang H. (2022). Bacterial nanocellulose-reinforced gelatin methacryloyl hydrogel enhances biomechanical property and glycosaminoglycan content of 3D-bioprinted cartilage. Int. J. Bioprinting.

[39] Hua S., Zhang H., Gu J., Yin M., Wu S., He C., Liu H., Zhou H., Guo R., Shi Y. (2026). Enhanced Auricular Cartilage Regeneration via 3D-Printed Hydrogel with miR-92a-3p-Enriched Platelet-Rich Plasma-Derived Extracellular Vesicles. Adv. Healthc. Mater..

[40] Landau S., Szklanny A.A., Machour M., Kaplan B., Shandalov Y., Redenski I., Beckerman M., Harari-Steinberg O., Zavin J., Karni-Katovitch O. (2021). Human-engineered auricular reconstruction (hEAR) by 3D-printed molding with human-derived auricular and costal chondrocytes and adipose-derived mesenchymal stem cells. Biofabrication.

[41] Zielinska D., Fisch P., Moehrlen U., Finkielsztein S., Linder T., Zenobi-Wong M., Biedermann T., Klar A.S. (2023). Combining bioengineered human skin with bioprinted cartilage for ear reconstruction. Sci. Adv..

[42] Jia L., Hua Y., Zeng J., Liu W., Wang D., Zhou G., Liu X., Jiang H. (2022). Bioprinting and regeneration of auricular cartilage using a bioactive bioink based on microporous photocrosslinkable acellular cartilage matrix. Bioact. Mater..

[43] Huang N., Lee S.K.J., Lee K.H.K., Wen C., Chang Y., Zhang M., Dong C., Mao C., Wang D.M., Ker D.F.E. (2026). Unconventional auricular reconstruction using controlled scaffold buckling and chondrogenic cocktail containing muscle-derived cells. Bioact. Mater..

[44] Zhang G., Li J., Wang H., Shangguan C., Xie J., Zhou Y. (2025). Research on the Impact Toughness of 3D-Printed CoCrMo Alloy Components Based on Fractal Theory. Biomimetics.

