# Supplementary material for: Cellularized Thermoformed Scaffolds with Human Septal Chondrocytes Support In Vivo Cartilage Maturation for Auricular Reconstruction
Source: J Funct Biomater. 2026 Sep 7;17(9):453. doi: 10.3390/jfb17090453 (PMC13608860; doi:10.3390/jfb17090453)
Supplement: Supplementary file 1 [file jfb-17-00453-s001.zip › Supplementary Table S1_Muinos-Lopez.pdf]

Table S1: Summary of cell's donors, characteristics, number of samples and experiments used in this work.

DONOR ID	DONOR SEX	DONOR AGE	NUMBER OF SAMPLES	TIME OF ANALYSIS	EXPERIMENT	MICE	TECHNIQUES	SCAFFOLD TYPE
NC1	Male	21 yo	2	7 days	IN VITRO	-	Life/dead & ECM synthesis	Squared
			2	28 days	IN VITRO	-	Life/dead & ECM synthesis	Squared
NC2	Female	61 yo	2	7 days	IN VITRO	-	Life/dead & ECM synthesis	Squared
			2	28 days	IN VITRO	-	Life/dead & ECM synthesis	Squared
IC1	Female	40 yo	2	7 days	IN VITRO	-	Life/dead & ECM synthesis	Squared
			2	28 days	IN VITRO	-	Life/dead & ECM synthesis	Squared
Empty (CTL without cells)	-	-	2	3 weeks	IN VIVO	1 (Female; 7 weeks)	Molecular Biology & Histology	Squared
			2	3 weeks			Mechanical Test	Squared
			2	6 weeks	IN VIVO	1 (Female; 7 weeks)	Molecular Biology & Histology	Squared
			2	6 weeks			Mechanical Test	Squared
			2	12 weeks	IN VIVO	1 (Female; 7 weeks)	Molecular Biology & Histology	Squared
			2	12 weeks			Mechanical Test	Squared
NC24	Female	16 yo	2	3 weeks	IN VIVO	1 (Female; 7 weeks)	Molecular Biology & Histology	Squared
			2	3 weeks			Mechanical Test	Squared
			2	6 weeks	IN VIVO	1 (Female; 7 weeks)	Molecular Biology & Histology	Squared

			2	6 weeks			Mechanical Test	Squared
			2	12 weeks	IN VIVO	1 (Female; 7 weeks)	Molecular Biology & Histology	Squared
			2	12 weeks			Mechanical Test	Squared
Empty (CTL without cells)	-	-	6	T0*	-	-	Mechanical Test	Squared
			1	6 weeks	IN VIVO	1 (Female; 6-10 weeks)	Molecular Biology & Histology	Squared
			2	6 weeks	IN VIVO	1 (Female; 6-10 weeks)	Mechanical Test	Squared
				12 weeks	IN VIVO	1 (Female; 6-10 weeks)	Molecular Biology & Histology	Squared
			3	12 weeks			Mechanical Test	Squared
NC25	Female	34 yo	6	T0*	-	-	Mechanical Test	Squared
			3	6 weeks	IN VIVO	1 (Female; 6-10 weeks)	Molecular Biology & Histology	Squared
			2	12 weeks	IN VIVO	3 (Female; 6-10 weeks)	Molecular Biology & Histology	Squared
			10	12 weeks			Mechanical Test	Squared
Empty (CTL without cells)	-	-	2	T0*	-	-	Mechanical Test	Squared
NC26	Male	21 yo	6	T0*	-	-	Mechanical Test	Squared
Empty (CTL without cells)	-	-	6	12 weeks	IN VIVO	2 (Female; 6-10 weeks)	Mechanical Test	Squared
			2	12 weeks	IN VIVO		Molecular Biology & Histology	Squared
NC10	Female	24 yo	3 Thermoformed Ear-	12 weeks	IN VIVO	1 (Female; 8 weeks) + 2	Molecular Biology, Histology,	Thermoformed Ear-shape

			shape samples (Each sample cutted in 4 pieces for different areas of analysis)			(Males; 7 weeks)	Immunofluorescence, Bulk RNA-seq	
NC19	Male	19 yo		12 weeks	IN VIVO		Molecular Biology, Histology, Immunofluorescence, Bulk RNA-seq	Thermoformed Ear-shape
NC25	Female	34 yo		12 weeks	IN VIVO		Molecular Biology, Histology, Immunofluorescence, Bulk RNA-seq	Thermoformed Ear-shape
NC32	Female	39 yo		12 weeks	IN VIVO		Molecular Biology, Histology, Immunofluorescence, Bulk RNA-seq	Thermoformed Ear-shape
NC37	Female	49 yo		12 weeks	IN VIVO		Molecular Biology, Histology, Immunofluorescence, Bulk RNA-seq	Thermoformed Ear-shape
NC16	Male	50 yo	1	Fresh cells from tissue digestion	-	-	Bulk RNA-seq	-

NC25	Female	34 yo	1	Fresh cells from tissue digestion	-	-	Bulk RNA-seq	-
NC26	Male	21 yo	1	Fresh cells from tissue digestion	-	-	Bulk RNA-seq	-
NC30	Male	38 yo	1	Fresh cells from tissue digestion	-	-	Bulk RNA-seq	-

NC = Nasal chondrocytes
IC = Intercostal chondrocytes
yo = Years old
*T0 = Time of scaffold combination (PLA-PEG+Alg with or without cells)

NOTE: Animals were randomly allocated to experimental groups (e.g., control vs. cellularized constructs, and different timepoints). All human cell samples and implanted construct conditions were fully anonymized prior to processing. Each retrieved sample after implantation was assigned a unique numerical code identifier, completely masking group identity during sample preparation, histological sectioning, mechanical testing, and molecular profiling.
